# MicroRNA‐381 in human cancer: Its involvement in tumour biology and clinical applications potential

**DOI:** 10.1111/jcmm.17161

**Published:** 2022-01-11

**Authors:** Huanhuan Sha, Yujie Gan, Feng Xu, Yue Zhu, Renrui Zou, Weiwei Peng, Zhiya Wu, Rong Ma, Jianzhong Wu, Jifeng Feng

**Affiliations:** ^1^ Department of Chemotherapy Jiangsu Cancer Hospital Jiangsu Institute of Cancer Research The Affiliated Cancer Hospital of Nanjing Medical University Nanjing Jiangsu China; ^2^ School of Basic Medical Sciences Xinxiang Medical University Xinxiang Henan China

**Keywords:** cancers, diagnosis, microRNA‐381, prognosis, therapy, tumour biology

## Abstract

MicroRNAs (miRNAs) are small non‐coding RNAs that regulate gene expression at the post‐transcriptional level. MiRNAs are involved in the development and progression of a wide range of cancers. Among such cancer‐associated miRNAs, miR‐381 has been a major focus of research. The expression pattern and role of miR‐381 vary among different cancer types. MiR‐381 modulates various cellular behaviours in cancer, including proliferation, apoptosis, cell cycle progression, migration and invasion. MiR‐381 is also involved in angiogenesis and lymphangiogenesis, as well as in the resistance to chemotherapy and radiotherapy. MiR‐381 itself is regulated by several factors, such as long noncoding RNAs, circular RNAs and cytokines. Aberrant expression of miR‐381 in blood samples indicates that it can be used as a diagnostic marker in cancer. Tissue miR‐381 expression may serve as a prognostic factor for the clinicopathological characteristics of cancers and survival of patients. Metformin and icaritin regulate miR‐381 expression and present anticancer properties. This review comprehensively summarizes the effect of miR‐381 on tumour biological behaviours, as well as the clinical application potential of miR‐381 for the treatment of cancer.

## 1 INTRODUCTION

MicroRNAs (miRNAs) are a novel class of endogenous, highly conserved, non‐coding RNAs of 18–25 nucleotides in length.^1^, ^2^ MiRNAs recognize and interact with the 3′‐untranslated region (3′‐UTR) of target mRNAs, thereby repressing gene expression post‐transcriptionally.^2^, ^3^ Abnormal expression of miRNAs is associated with the occurrence of various diseases, especially cancers. Extensive research efforts have been dedicated to select eligible miRNAs as credible markers for cancer diagnosis and valuable targets for cancer therapy.

MiR‐381 has become a research hotspot among cancer‐related miRNAs. The gene encoding miR‐381 is located in human chromosome band 14q32 and its transcription generates pre‐miR‐381.^4^, ^5^ MiR‐381 exerts its function in cancer by regulating cell proliferation, apoptosis, cell cycle progression, migration and invasion. Lymphangiogenesis and tumour angiogenesis are also regulated by miR‐381. Here, we systematically reviewed the role of miR‐381 in cancer and the underlying mechanisms to highlight the potential of miR‐381 as a biomarker for cancer diagnosis and a target for cancer therapy.

## 2 THE EXPRESSION OF MIR‐381 IN CANCERS

The expression patterns of miR‐381, including downregulation and upregulation in different cancer types, have been investigated and reported (Table 1). Expression of the miR‐381 gene is decreased in diffuse large B cell lymphoma (DLBCL)^6^ and non‐small cell lung cancer (NSCLC).^5^, ^7^, ^8^, ^9^ MiR‐381 is also downregulated in multiple cancers related to the digestive system, including oral squamous cell carcinoma (OSCC), oesophageal squamous cell carcinoma (ESCC), gastric cancer, colorectal cancer (CRC), hepatocellular carcinoma (HCC) and pancreatic cancer,^10^, ^11^, ^12^, ^13^, ^14^, ^15^, ^16^, ^17^, ^18^, ^19^ as well in urogenital neoplasms, such as renal cell carcinoma (RCC), bladder cancer, prostate cancer (PCa), ovarian cancer, endometrial cancer (EMC) and cervical cancer.^20^, ^21^, ^22^, ^23^, ^24^, ^25^, ^26^, ^27^, ^28^, ^29^ Low expression of the miR‐381 gene is also found in breast cancer, papillary thyroid cancer (PTC), basal cell carcinoma (BCC) and laryngeal squamous cell carcinoma (LSCC).^30^, ^31^, ^32^, ^33^, ^34^, ^35^, ^36^, ^37^

**TABLE 1:** MiR‐381 expression in human cancers

System	Type	Expression	Level	Reference
Haemolymphatic system	DLBCL	Down	Tissue	[6]
Nervous system	Glioma	Up	Tissue, cell and blood	[38, 43]
Respiratory system	NSCLC	Down	Tissue and cell	[5, 7, 8, 9]
Digestive system	OSCC	Down	Tissue and cell	[10]
	ESCC	Down	Tissue and cell	[11]
	Gastric cancer	Down	Tissue, cell and serum	[12, 13, 14, 15, 16, 84]
	CRC	Down	Tissue and cell	[17]
	HCC	Down	Tissue and cell	[18]
	Pancreatic cancer	Down	Tissue and cell	[19]
Urinary system	RCC	Down	Tissue and cell	[20, 21]
	Bladder cancer	Down	Tissue and cell	[22]
Reproductive system	PCa	Down	Tissue and cell	[23, 24]
		Up	Plasma	[85]
	Ovarian cancer	Down	Tissue, cell and serum	[25, 26]
	EMC	Down	Tissue and cell	[27]
	Cervical cancer	Down	Tissue and cell	[28, 29]
Musculoskeletal system	Osteosarcoma	Up	Tissue	[39]
	Synovial sarcoma	Up	Tissue	[40]
	Epithelioid sarcoma	Up	Tissue	[41]
Others	Breast cancer	Down	Tissue and cell	[30, 31, 32, 33, 34]
	PTC	Down	Tissue and cell	[35]
	BCC	Down	Tissue	[36]
	LSCC	Down	Tissue and cell	[37]

Abbreviations: BCC, basal cell carcinoma; CRC, colorectal cancer; DLBCL, diffuse large B cell lymphoma; EMC, endometrial cancer; ESCC, oesophageal squamous cell carcinoma; HCC, hepatocellular carcinoma; LSCC, laryngeal squamous cell carcinoma; NSCLC, non‐small cell lung cancer; OSCC, oral squamous cell carcinoma; PCa, prostate cancer; PTC, papillary thyroid cancer; RCC, renal cell carcinoma.

By contrast, miR‐381 is overexpressed in glioma^38^ and in musculoskeletal tumours including osteosarcoma, synovial sarcoma and epithelioid sarcoma.^39^, ^40^, ^41^

## 3 THE BIOLOGICAL ROLE OF MIR‐381 IN HUMAN CANCERS

### 3.1 Malignancies of haematologic and lymphatic systems

#### 3.1.1 Diffuse large B cell lymphoma

Approximately 30–40% of patients with DLBCL have primary refractory disease or experience relapse with a poor prognosis. Compared with primary DLBCL, relapsed DLBCL is characterized by decreased miR‐381 expression. MiR‐381 increases the sensitivity of DLBCL to both doxorubicin and rituximab and may sensitize DLBCL to chemotherapy and targeted drugs through the downregulation of inositol monophosphatase 1 and phosphoinositide 3‐ kinase catalytic subunit delta (PI3KCD) (Table 2).^6^

**TABLE 2:** Biological role of miR‐381 in haematolymphoid malignancies

Cancer type	Direct target	Downstream signal	Effect	Reference
DLBCL	Undetermined	IMP‐1/PI3KCD	Enhances sensitivity to chemotherapy and targeted therapy	[6]
CML	MDR‐1	Undetermined	Inhibits drug efflux, promotes drug intracellular accumulation and reverses chemoresistance	[42]

Abbreviations: CML, chronic myeloid leukaemia; DLBCL, diffuse large B cell lymphoma; IMP‐1, inositol monophosphatase 1; MDR‐1, multidrug resistance gene 1; PI3KCD, phosphoinositide 3‐ kinase, catalytic subunit delta.

#### 3.1.2 Chronic myeloid leukaemia

MiR‐381 expression is lower in chemoresistant Chronic myeloid leukaemia (CML) cells than in parental cells, suggesting that it acts as a tumour suppressor by interfering with chemoresistance (Table 2). Mechanistically, miR‐381 resensitizes doxorubicin‐resistant and vinblastin‐resistant CML cells by targeting the multidrug resistance gene 1 (MDR‐1). MiR‐381 downregulates the expression of P‐glycoprotein (P‐gp), the protein product of the MDR‐1 gene, thereby inhibiting drug efflux in chemoresistant cells and leading to the intracellular accumulation of chemotherapeutic drugs.^42^

### 3.2 Glioma

MiR‐381 is overexpressed in glioma and may function as an oncomiR (Table 3). The expression of miR‐381 is negatively correlated with that of leucine‐rich repeat containing‐4 (LRRC‐4).^43^ MiR‐381 targets LRRC‐4, thereby suppressing the expression of bromodomain‐containing protein 7 (BRD7), a transcriptional cofactor for the tumour suppressor gene p53, and promoting cell proliferation in glioma. The proliferation‐promotive activity of miR‐381‐induced suppression of LRRC‐4 is mediated by the inactivation of the Ras/Raf/extracellular regulated protein kinase (ERK) pathway and the phosphoinositide 3‐kinase (PI3K)/protein kinase B (PKB/Akt) pathway.^44^ Neurofilament light polypeptide (NEFL) is also a target of miR‐381 in glioma. MiR‐381 downregulates NEFL and induces the expression of MDR proteins including ATP‐binding cassette subfamily C member 3 (ABCC3), ABCC5 and ABCG2, as well as the expression of stemness markers such as acetaldehyde dehydrogenase 1, cluster of differentiation 44, C‐KIT, Kruppel‐like factor 4, the homeobox protein Nanog, Nestin and the transcription factor SOX2. The upregulation of MDR and stemness factors by miR‐381 confers glioma cells resistance to temozolomide. The oncogenic role of miR‐381 may also be attributed to the inactivation of the mammalian target of rapamycin (mTOR) pathway via the suppression of NEFL.^38^

**TABLE 3:** Biological role of miR‐381 in glioma

Cancer type	Direct target	Downstream signal	Effect	Reference
Glioma	LRRC‐4	BRD7—P53 pathway/Ras—Raf—ERK pathway/ PI3K—Akt pathway	Promotes proliferation	[43, 44]
	NEFL	ABCC3/ABCC5/ABCG2/ALDH1/CD44/C‐KIT/KLF4/Nanog/Nestin/SOX2/mTOR pathway	Promotes proliferation, invasion and induces chemoresistance	[38]

Abbreviations: ABCC3, ATP‐binding cassette subfamily C member 3; ABCC5, ATP‐binding cassette subfamily C member 5; ABCG2, ATP‐binding cassette subfamily G member 2; Akt, protein kinase B; ALDH1, acetaldehyde dehydrogenase 1; BRD7, bromodomain‐containing protein 7; CD44, cluster of differentiation 44; ERK, extracellular regulated protein kinases; KLF4, krueppel‐like factor 4; LRRC‐4, leucine‐rich repeat containing‐4; mTOR, mammalian target of rapamycin; NEFL, neurofilament light polypeptide; PI3K, phosphoinositide 3‐kinase.

### 3.3 Non‐small cell lung cancer

The downregulation of miR‐381 indicates its suppressive role in NSCLC (Table 4). MiR‐381 targets LIM domain only protein 3 (LMO3) and negatively regulates the PI3K/Akt signalling pathway, suppressing cell proliferation in NSCLC. The decrease of LMO3 downregulates N‐cadherin and vimentin and upregulates E‐cadherin, thus reversing the epithelial‐mesenchymal transition (EMT) process in NSCLC. The reversal of EMT by miR‐381 results in the inhibition of the migratory and invasive abilities of NSCLC cells. In addition, miR‐381 targets Snail and Twist‐related protein 1 (Twist1), which are classical EMT inducers, thereby reversing the EMT process and repressing the migratory and invasive capabilities of NSCLC cells.^5^, ^45^ The inhibition of yes‐associated protein (YAP) by miR‐381 causes the reversal of EMT and the suppression of cell migration and invasion in NSCLC.^9^ Liver receptor homologue 1 (LRH‐1) is a novel oncogene in several cancers.^46^ MiR‐381 suppresses the expression of LRH‐1 in NSCLC, which inhibits cell migration and invasion.^7^ The downregulation of inhibitor of differentiation 1 (ID‐1) is responsible for the suppressive role of miR‐381 in NSCLC cells as well.^8^ MiR‐381 inhibits ID‐1, thereby suppressing the activation of nuclear factor kappa‐light‐chain‐enhancer of activated B (NF‐κB) and inhibiting the expression of both B‐cell leukaemia/lymphoma‐2 (Bcl‐2) and Bcl‐XL, thus suppressing cell proliferation and inducing cell apoptosis in NSCLC, respectively. Furthermore, miR‐381 reverses cisplatin resistance in NSCLC.^47^ In addition to its effect on the resistance to traditional chemotherapy drugs, miR‐381 also affects the resistance of NSCLC to immunotherapy, such as anti‐programmed cell death protein 1 (PD‐1)‐based therapy. MiR‐381 targets C‐X‐C motif chemokine receptor 4 (CXCR4) and reverses the resistance of NSCLC to anti‐PD‐1‐based therapy.^48^ Emerging evidence supports the suppressive effect of miR‐381 on cell cycle progression in NSCLC. Huang et al. reported that miR‐381 may arrest the cell cycle at the G0/G1 phase by upregulating p21 and p27 and downregulating cyclin D1 and cyclin‐dependent kinase 4 (CDK4).^47^ However, another investigation from Guo et al. suggested that miR‐381 induces G2 phase arrest by suppressing ubiquitin‐conjugating enzyme E2C (UBE2C). Moreover, miR‐381 may activate autophagy by targeting UBE2C, thus reversing the EMT process and inhibiting cell migration and invasion in NSCLC. MiR‐381‐induced autophagy also limits cell proliferation and promotes cell apoptosis in NSCLC.^49^

**TABLE 4:** Biological role of miR‐381 in NSCLC

Cancer type	Target	Downstream signal	Effect	Reference
NSCLC	LMO3	PI3K—Akt pathway	Inhibits proliferation, reverse EMT phenotype and inhibits migration and invasion	[5]
	Twist1/Snail	Undetermined	Reverse EMT phenotype, inhibits migration and invasion	[45]
	YAP	Undetermined	Inhibits proliferation, reverse EMT phenotype and inhibits migration and invasion	[9]
	LRH‐1	Undetermined	Inhibits migration and invasion	[7]
	ID1	NF‐κB pathway/Bcl‐2/Bcl‐xL	Inhibits proliferation, promotes apoptosis and reverses chemoresistance	[8]
	CXCR4	Undetermined	Inhibits proliferation, invasion, immune evasion and reverses resistance to anti‐PD‐1 therapy	[48]
	Undetermined	p21/p27—cyclin D1—CDK4 pathway	Arrests cell cycle at G0/G1 phase	[47]
	UBE2C	Autophagy	Arrests cell cycle at G2 phase, reverses EMT phenotype, inhibits migration, invasion, proliferation and promotes apoptosis	[49]

Abbreviations: Akt, protein kinase B; Bcl‐2, B‐cell leukaemia/lymphoma‐2; Bcl‐XL, B‐cell leukaemia/lymphoma‐2 XL; CDK4, cyclin‐dependent kinase 4; CXCR4, C‐X‐C motif chemokine receptor 4; EMT, epithelial–mesenchymal transition; ID1, inhibitor of differentiation 1; LMO3, LIM domain only protein 3; LRH‐1, liver receptor homologue 1; NF‐κB, nuclear factor kappa‐light‐chain‐enhancer of activated B cells; NSCLC, non‐small cell lung cancer; PD‐1, programmed cell death protein 1; PI3K, phosphoinositide 3‐kinase; Twist1, Twist‐related protein 1; UBE2C, ubiquitin‐conjugating enzyme E2C; YAP, yes‐associated protein.

### 3.4 Digestive cancers

#### 3.4.1 OSCC and ESCC

With more than 30,000 new cases diagnosed each year, OSCC accounts for approximately 90% of all oral cancers.^50^, ^51^ MiR‐381 serves as a tumour suppressor in OSCC (Table 5). MiR‐381 directly targets fibroblast growth factor receptor 2 (FGFR2), restraining cell proliferation and blocking cell cycle progression at the G0/G1 phase. Inhibition of FGFR2 promotes cell apoptosis in OSCC.^10^ Similar tumour‐suppressive properties were observed in ESCC, where miR‐381 targets X‐linked inhibitor of apoptosis protein, thereby contributing to the reversal of radioresistance in ESCC.^11^

**TABLE 5:** Biological role of miR‐381 in digestive cancers

Cancer type	Target	Downstream signal	Effect	Reference
OSCC	FGFR2	Undetermined	Inhibits proliferation, arrests cell cycle at G0/G1 phase and promotes apoptosis	[10]
ESCC	XIAP	Undetermined	Inhibits proliferation, promotes apoptosis and reverses radioresistance	[11]
Gastric cancer	Twist1	Undetermined	Inhibits migration, invasion and promotes apoptosis	[15]
	SOX4	Undetermined	Reverses EMT phenotype, inhibits migration and invasion	[16]
	ROCK2	Undetermined	Inhibits proliferation, migration and invasion	[14]
	TMEM16A	TGF‐β pathway	Inhibits migration and invasion	[13]
	CUL4B	Wnt—β‐catenin pathway	Reverses EMT phenotype, inhibits migration and invasion	[12]
	ZEB1	Wnt—β‐catenin pathway, JNK pathway	Inhibits proliferation, migration, invasion and promotes apoptosis	[53]
CRC	Twist1	Undetermined	Reverses EMT phenotype, inhibits migration, invasion and proliferation	[17]
	UBE2C	Undetermined	Inhibits proliferation, migration and promotes apoptosis	[54]
Pancreatic cancer	CXCR4	Undetermined	Inhibits proliferation, migration and invasion	[55]
	EST1	PI3K—Akt—mTOR pathway	Arrests cell cycle at G1 phase, inhibits proliferation, migration, invasion and promotes apoptosis	[19]
HCC	LRH‐1	Wnt pathway	Arrests cell cycle at G0/G1 phase, inhibits proliferation and invasion	[18]

Abbreviations: Akt, protein kinase B; CUL4B, Cullin 4B; CXCR4, C‐X‐C motif chemokine receptor 4; EMT, epithelial–mesenchymal transition; ESCC, oesophageal squamous cell carcinoma; EST1, ever shorter telomeres protein 1; FGFR2, fibroblast growth factor receptor 2; HCC, hepatocellular carcinoma; JNK, c‐Jun N‐terminal kinase; LRH‐1, liver receptor homologue 1; mTOR, mammalian target of rapamycin; OSCC, oral squamous cell carcinoma; PI3K, phosphoinositide 3‐kinase; ROCK2, Rho‐associated coiled‐coil containing protein kinase 2; SOX4, SRY‐Box Transcription Factor 4; TGF‐β, transforming growth factor‐β; TMEM16A, transmembrane protein 16A; Twist1, Twist‐related protein 1; UBE2C, ubiquitin‐conjugating enzyme E2C; XIAP, X‐linked inhibitor of apoptosis protein; ZEB1, zinc finger E‐box‐binding homeobox 1.

#### 3.4.2 Gastric cancer

In gastric cancer, miR‐381 exerts a tumour suppressive role (Table 5). It targets Twist1 and suppresses cell proliferation and invasion, as well as promoting cell apoptosis.^15^ The SRY‐box transcription factor 4 (SOX4) gene is overexpressed in >20 types of cancer. In gastric cancer, miR‐381 reverses the EMT process and thus limits the migratory and invasive capacities of gastric cancer cells by downregulating SOX4 expression.^16^ MiR‐381 targets Rho‐associated coiled‐coil containing protein kinase 2 (ROCK2), which plays a role in the organization of the actin cytoskeleton and the progression of many cancers, thereby suppressing cell proliferation, migration and invasion in gastric cancer.^14^ The transforming growth factor‐β (TGF‐β) signalling pathway plays a dual role in cancer development. In pre‐malignant cells, TGF‐β suppresses cell proliferation and enhances cell apoptosis. However, in the later phase, TGF‐β induces the EMT phenotype and promotes tumour metastasis.^52^ In gastric cancer, miR‐381 represses the secretion of TGF‐β by targeting transmembrane protein 16A. Moreover, the synthesis of TGF‐β may also be suppressed by miR‐381. The inhibition of TGF‐β signalling further reverses the EMT process, leading to the inhibition of cell migration and invasion in gastric cancer.^13^ The involvement of the Wnt/β‐catenin pathway in carcinogenesis in various malignancies is well documented. In gastric cancer, the Wnt/β‐catenin pathway is highly activated. MiR‐381 directly targets Cullin 4B (CUL4B), an oncogene overexpressed in various cancers, and downregulates the expression of β‐catenin as well as c‐MYC and cyclin D1 in gastric cancer cells. The repression of the Wnt/β‐catenin pathway accounts for the inhibitory effect of miR‐381 on the EMT process and on cell migration and invasion in gastric cancer.^12^ MiR‐381 may also regulate the Wnt/β‐catenin pathway by downregulating zinc finger E‐box binding homeobox 1 (ZEB1); this suppresses the phosphorylation of c‐Jun N‐terminal kinase (JNK) and c‐Jun and decreases the expression of Wnt3a, Wnt5a and β‐catenin in gastric cancer cells. The inactivation of the JNK and Wnt/β‐catenin pathways mediated by miR‐381 ultimately leads to the suppression of cell proliferation, migration and invasion along with the acceleration of cell apoptosis in gastric cancer.^53^

##### Colorectal cancer

Similar to its role in gastric cancer, miR‐381 may also act as a negative regulator of Twist1 in CRC (Table 5). MiR‐381 directly targets Twist1 and reverses the EMT phenotype, restricting cell migration and invasion in CRC. The downregulation of Twist1 also inhibits the proliferation of CRC cells.^17^ Aberrant upregulation of UBE2C is observed in CRC, and the expression of UBE2C is modulated by miR‐381 (Table 5). MiR‐381 downregulates UBE2C, thereby decreasing cell proliferation and migration and promoting cell apoptosis, thus exerting a suppressive effect in CRC.^54^

#### 3.4.3 Pancreatic cancer

The network of chemokines and receptors in the tumour microenvironment is complex. Chemokines interact with relevant receptors and have various biological functions in cancer. In pancreatic cancer, CXCR4 is a target of miR‐381 and its expression is upregulated. Under stimulation by its functional ligand CXCL12, CXCR4 not only promotes cell survival and proliferation but also facilitates cell motility and invasion in pancreatic cancer (Table 5).^55^ The carcinostatic action of miR‐381 in pancreatic cancer may also be attributed to its inhibitory effect on the expression of ever shorter telomeres protein 1 (EST1) (Table 5). MiR‐381 also blocks cell cycle progression by arresting pancreatic cells at the G1 phase. Furthermore, the effect of miR‐381 on modulating cellular behaviours in pancreatic cancer might be dependent on the inactivation of the PI3K/Akt/mTOR signalling pathway via EST1 suppression.^19^

#### 3.4.4 Hepatocellular carcinoma

LRH‐1 acts as an oncogene in HCC by modulating cell proliferation and apoptosis, thereby promoting the tumourigenic potential of HCC cells.^56^, ^57^ LRH‐1 may also increase hepatitis B virus DNA replication and gene transcription, which increases the tumourigenesis of HCC.^58^ MiR‐381 regulates the expression of LRH‐1 in HCC (Table 5) and negatively modulates the transcriptional activity of the Wnt pathway. The downregulation of cyclin D1, cyclin E1 and matrix metalloproteinase 9, which are target genes of the Wnt pathway, may underlie the inhibition of cell proliferation and invasion and the induction of G0/G1 phase arrest by miR‐381 in HCC.^18^

### 3.5 Urogenital cancers

#### 3.5.1 Renal cell carcinoma

Renal cell carcinoma is highly malignant and insensitive to chemotherapy and radiotherapy. MiR‐381 is downregulated in RCC, supporting its tumour‐suppressive role (Table 6). MiR‐381 directly targets regulatory factors critical for tumour invasion and metastasis, such as CREB‐binding protein, β‐catenin and lymphoid enhancer‐binding factor‐1, suppressing cell migration and invasion in RCC.^59^ In addition, it targets Wee1‐like protein kinase (WEE1) and inhibits its activation, thereby inhibiting the suppressive effect of WEE1 on cell‐division‐cycle kinase 2 (CDC2), which is a critical mitotic inducer and a regulator of cell proliferation, apoptosis and cell cycle progression.^60^ MiR‐381 increases CDC2 activity, thus inhibiting cell proliferation and promoting cell apoptosis in RCC in a synergistic manner.^21^ WEE1 depletion‐mediated activation of CDC2 is also responsible for the sensitization of RCC cells to 5‐fluorouracil induced by miR‐381. MiR‐381 reverses the resistance of RCC to other chemotherapy drugs by suppressing cell proliferation and promoting cell apoptosis, which sensitizes RCC to cisplatin and paclitaxel.^20^

**TABLE 6:** Biological role of miR‐381 in urogenital cancers

Cancer type	Target	Downstream signal	Effect	Reference
RCC	CBP/β‐catenin/LEF‐1	Undetermined	Inhibits migration and invasion	[59]
	WEE1	CDC2	Inhibits proliferation, promotes apoptosis, and enhances sensitivity to chemotherapy	[60]
Bladder cancer	cyclin A2/CDK6	Rb	Arrests cell cycle at G1 stage, inhibits proliferation	[22]
	cyclin A2	ROCK—Snail	Reverses EMT phenotype, inhibits migration	[22]
	MET	Akt—GSK‐3β—Snail	Reverses EMT phenotype, inhibits migration	[22]
PCa	AR	Wnt—β‐catenin pathway	Inhibits proliferation, migration, invasion and promotes apoptosis	[23]
	UBE2C	Undetermined	Inhibits proliferation and invasion	[63]
Ovarian cancer	YY1	Undetermined	Inhibits proliferation, migration and invasion	[26]
	PIK3CA	Undetermined	Inhibits proliferation, migration and invasion	[25]
EMC	IGF‐1R	ERK/Akt	Inhibits proliferation and invasion	[27]
Cervical cancer	FGF‐7	Undetermined	Arrests cell cycle at G0/G1 phase, inhibits migration, invasion and promotes apoptosis	[28]
	HOXA13	Undetermined	Inhibits proliferation and invasion	[29]

Abbreviations: Akt, protein kinase B; AR, androgen receptor; CBP, CREB‐binding protein; CDC2, cell‐division‐cycle kinase 2; CDK6, cyclin‐dependent kinase; EMC, endometrial cancer; EMT, epithelial–mesenchymal transition; ERK, extracellular‐regulated protein kinases; FGF‐7, fibroblast growth factor 7; GSK‐3β, glycogen synthase kinase‐3β; HOXA13, homeobox A13; IGF‐1R, insulin‐like growth factor 1 receptor; LEF‐1, lymphoid enhancer binding factor‐1; PCa, prostate cancer; PIK3CA, phosphatidylinositol‐4, 5‐bisphosphate 3‐kinase catalytic subunit alpha; Rb, retinoblastoma tumour suppressor protein; RCC, renal cell carcinoma; ROCK, Rho‐associated coiled‐coil containing protein kinase; UBE2C, ubiquitin‐conjugating enzyme E2C; WEE1, Wee1‐like protein kinase; YY1, Yin Yang‐1.

#### 3.5.2 Bladder cancer

Bladder cancer is the most common urologic malignancy, and it is associated with an unfavourable prognosis.^61^ In bladder cancer, miR‐381 interferes with cell cycle progression (Table 6). It directly targets cyclin A2 and cyclin‐dependent kinase 6 (CDK6), thereby inhibiting the phosphorylation of retinoblastoma protein (Rb). The suppression of Rb phosphorylation by miR‐381 blocks cell cycle progression and induces G1 phase arrest. In addition, miR‐381 suppresses cyclin A2, which downregulates ROCK and negatively regulates the expression of Snail, an EMT inducer (Table 6). The downregulation of Snail may also be attributed to the direct repression of MET by miR‐381 and the subsequent inactivation of Akt and glycogen synthase kinase‐3β. Inhibition of Snail by miR‐381 reverses the EMT phenotype and decreases cell migration in bladder cancer (Table 6).^22^

#### 3.5.3 Prostate cancer

Androgen deprivation therapy (ADT) is regarded as the first therapeutic option for PCa. However, the efficacy of ADT is limited because most cases become resistant to therapy within 2 years.^62^ The androgen receptor (AR) is activated by numerous factors in addition to androgen. Therefore, blockade of AR signalling is a promising therapy for refractory PCa. A recent study by Rui et al. identified miR‐381 as a negative regulator of AR signalling (Table 6). In PCa, miR‐381 targets AR and downregulates its expression, thereby playing an inhibitory role in PCa.^23^ In addition, downregulation of UBE2C mediated by miR‐381 is responsible for reduced cell proliferation and invasion in PCa^63^ (Table 6).

#### 3.5.4 Ovarian cancer

In developed countries, ovarian cancer accounts for the majority of deaths among all gynecologic malignancies.^64^ MiR‐381 suppresses YY‐1, which decreases cell proliferation, migration and invasion in ovarian cancer.^26^ MiR‐381 may also act as the upstream regulator of phosphatidylinositol‐4, 5‐bisphosphate 3‐kinase catalytic subunit alpha (PIK3CA) (Table 6). MiR‐381 directly targets PIK3CA and negatively regulates cell proliferation, migration and invasion in ovarian cancer.^25^

#### 3.5.5 Endometrial cancer

Endometrial cancer is the sixth most common malignancy among women.^65^ MiR‐381plays a suppressive role in EMC (Table 6). It targets insulin like growth factor 1 receptor (IGF‐1R) and inhibits cell proliferation and invasion in EMC. Moreover, the suppressive role of miR‐381‐mediated IGF‐1R suppression is due to the dephosphorylation of ERK and Akt.^27^

#### 3.5.6 Cervical cancer

Human papillomavirus (HPV) infection is the leading cause of cervical cancer. Studies investigating the relation between HPV and fibroblast growth factor 7 (FGF‐7) suggest that FGF‐7 is involved in the progression of HPV.^66^, ^67^ FGF‐7 is upregulated in cervical cancer and may serve as the target of miR‐381 (Table 6). The downregulation of FGF‐7 by miR‐381 impedes cell cycle progression and arrests cells at the G0/G1 phase.^28^ MiR‐381 also inhibits the proliferative and invasive capabilities of cervical cancer cells by targeting HOXA13^29^ (Table 6).

### 3.6 Musculoskeletal malignancies

#### 3.6.1 Osteosarcoma

The role of miR‐381 in osteosarcoma remains controversial (Table 7). Xia et al.^68^ suggested that miR‐381 inactivates the JNK and Wnt/β‐catenin pathways by reversely targeting ZEB1. The anticancer activity of miR‐381 in osteosarcoma was validated by Tsai et al.,^69^ who showed that miR‐381 suppresses angiogenesis by targeting vascular endothelial growth factor‐A (VEGF‐A) and subsequently inhibiting the migration of endothelial progenitor cells (EPCs) as well as tube formation. Contrary to its suppressive role, miR‐381 also functions as an oncomiR in osteosarcoma. Mechanistically, miR‐381‐mediated suppression of LRRC‐4 induces cisplatin resistance by inactivating the mTOR pathway. Moreover, miR‐381‐mediated downregulation of LRRC‐4 decreases the expression of multidrug resistance‐associated proteins including ATP‐Binding Cassette Subfamily C Member 1 (ABCC1), ABCC2 and ABCG1, along with cluster of differentiation 133, a marker of cancer stem cells.^39^

**TABLE 7:** Biological role of miR‐381 in musculoskeletal malignancies

Cancer type	Target	Downstream signal	Effect	Reference
Osteosarcoma	ZEB1	JNK pathway/Wnt—β‐catenin pathway	Inhibits proliferation, migration, invasion and promotes apoptosis	[68]
	VEGF‐A	Undetermined	Inhibits EPC migration, tube formation and tumour angiogenesis	[69]
	LRRC‐4	mTOR pathway/ABCC1/ABCC2/ABCG1/CD133	promotes proliferation, invasion and induces chemoresistance	[39]
Chondrosarcoma	VEGF‐C	Undetermined	Inhibits LEC migration, tube formation and tumour lymphangiogenesis	[70]

Abbreviations: ABCC1, ATP‐Binding Cassette Subfamily C Member 1; ABCC2, ATP‐Binding Cassette Subfamily C Member 2; ABCG1, ATP‐Binding Cassette Subfamily G Member 1; CD133, cluster of differentiation 133; EPC, endothelial progenitor cell; JNK, c‐Jun N‐terminal kinase; LEC, lymphatic endothelial cell; LRRC‐4, leucine‐rich repeat containing‐4; mTOR, mammalian target of rapamycin; VEGF‐A, vascular endothelial growth factor‐A; VEGF‐C, vascular endothelial growth factor‐C; ZEB1, zinc finger E‐box‐binding homeobox 1.

#### 3.6.2 Chondrosarcoma

Tzeng et al. suggested that miR‐381 functions as a suppressor of lymphatic metastasis in chondrosarcoma (Table 7). MiR‐381 targets VEGF‐C, an enhancer of lymphangiogenesis, and represses tube formation and migration of lymphatic endothelial cells, thereby inhibiting lymphangiogenesis in chondrosarcoma.^70^

### 3.7 Breast cancer

MiR‐381 serves as a tumour suppressor in breast cancer (Table 8). It directly targets nicotinamide phosphoribosyltransferase and inhibits the expression of nicotinamide adenine dinucleotide, a crucial coenzyme for redox reactions in cancer cells, thereby decreasing cell proliferation and promoting cell apoptosis in breast cancer.^33^ The anti‐proliferative activity of miR‐381 may also be attributed to the suppression of CXCR‐4. MiR‐381‐induced downregulation of CXCR‐4 reverses the EMT process and suppresses cell migration and invasion in breast cancer as well.^34^ Ming et al. identified miR‐381 as the functional suppressor of connexin 43 (Cx43), which potentiates the migratory activity of breast cancer cells. Mechanistically, miR‐381 directly targets CCAAT‐enhancer‐binding protein‐α (C/EBPα), which upregulates the expression of Cx43 via direct binding to its promoter region.^71^ MiR‐381 also targets SET domain bifurcated 1, an oncogene in multiple cancers, causing cell cycle arrest at the G0/G1 phase and suppressing cell proliferation and migration in breast cancer.^72^ In triple‐negative breast cancer (TNBC), a highly aggressive subtype with poor prognosis, miR‐381 targets several n genes of the canonical and noncanonical Wnt pathways including β‐catenin, RhoA, ROCK1 and c‐MYC. The inhibition of these genes by miR‐381 decreases cell proliferation, migration and invasion in TNBC.^31^

**TABLE 8:** Biological role of miR‐381 in breast cancer

Cancer type	Target	Downstream signal	Effect	Reference
Breast cancer	NAMPT	NAD	Inhibits proliferation and promotes apoptosis	[33]
	CXCR‐4	Undetermined	Inhibits proliferation, reverses EMT phenotype and inhibits migration and invasion	[34]
	C/EBPα	Cx43	Inhibits migration	[71]
	SETDB1	Undetermined	Arrests cell cycle at G0/G1 phase, inhibits proliferation and migration	[72]
	Β‐ catenin/RhoA/ROCK1/c‐MYC	Undetermined	Inhibits proliferation, migration and invasion	[31]
	MDR‐1	Undetermined	Inhibits proliferation, promotes apoptosis and reverses chemoresistance	[30]
	FYN	p38/ERK	Inhibits proliferation, promotes apoptosis and enhances sensitivity to chemotherapy	[73]
	JARID1B	BRCA1	Arrests cell cycle at G0/G1 phase, inhibits proliferation and enhances sensitivity to radiotherapy	[32]

Abbreviations: BRCA1, breast cancer 1; C/EBPα, CCAAT‐enhancer‐binding protein‐α; Cx43, connexin 43; CXCR‐4, C‐X‐C motif chemokine receptor 4; EMT, epithelial–mesenchymal transition; ERK, extracellular regulated protein kinases; JARID1B, jumonji AT‐rich interactive domain 1B; MDR‐1, multidrug resistance gene 1; NAD, nicotinamide adenine dinucleotide; NAMPT, nicotinamide phosphoribosyltransferase; ROCK1, Rho‐associated coiled‐coil containing protein kinase 1; SETDB1, SET domain bifurcated 1.

The regulatory effect of miR‐381 on several cellular biological behaviours increases the sensitivity of breast cancer cells to chemotherapy and radiotherapy. MiR‐381 targeting of MDR‐1 reverses cisplatin‐resistance in breast cancer.^30^ In addition, miR‐381 directly targets FYN and suppresses the activation of p38 and ERK. The inactivation of the mitogen‐activated protein kinase signalling pathway further inhibits proliferation and induces apoptosis, thus enhancing the sensitivity of breast cancer to doxorubicin.^73^ The enhancement of radiosensitivity by miR‐381 in breast cancer is ascribed to the suppression of Jumonji AT rich interactive domain 1B (JARID1B). MiR‐381 targets JARID1B and promotes the expression of the tumour suppressor breast cancer 1, contributing to G0/G1 phase arrest as well as the suppression of cell proliferation in breast cancer.^32^

### 3.8 Other malignancies

#### 3.8.1 Papillary thyroid cancer

Papillary thyroid cancer accounts for approximately 70% cases of thyroid cancer.^74^ MiR‐381 serves as a tumour suppressor in PTC (Table 9). The anticarcinogenic effect of miR‐381 on PTC is attributed to the suppression of low‐density lipoprotein receptor‐related protein 6, an essential Wnt co‐receptor, to activate Wnt/β‐catenin signalling.^35^

**TABLE 9:** Biological role of miR‐381 in other cancer types

Cancer type	Target	Downstream signal	Effect	Reference
PTC	LRP6	Undetermined	Inhibits proliferation, migration and invasion	[35]
LSCC	NASP	Undetermined	Inhibits proliferation, reverses EMT phenotype and inhibits migration and invasion	[37]

Abbreviations: EMT, epithelial–mesenchymal transition; LRP6, low‐density lipoprotein receptor‐related protein 6; LSCC, laryngeal squamous cell carcinoma; NASP, nuclear autoantigenic sperm protein; PTC, papillary thyroid cancer.

#### 3.8.2 Laryngeal squamous cell carcinoma

In addition to OSCC, the suppressive role of miR‐381 was observed in other head‐neck squamous cell carcinomas such as LSCC. In LSCC, miR‐381 downregulates nuclear autoantigenic sperm protein (NASP) (Table 9), thereby decreasing cell proliferation and reversing the EMT phenotype, which reduces cell migration and invasion in LSCC.^37^

## 4 REGULATORY FACTORS OF MIR‐381 IN HUMAN CANCERS

### 4.1 LncRNA and CircRNA

**FIGURE 1:**
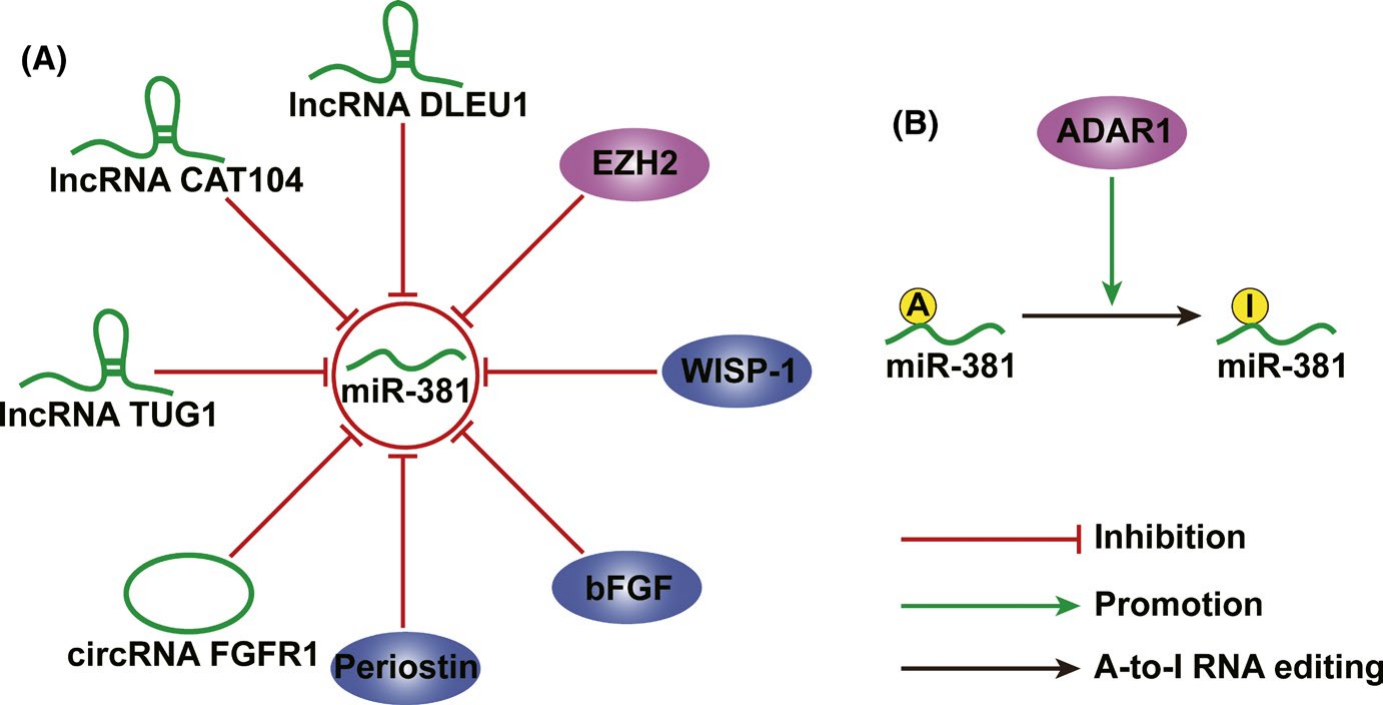
Regulatory factors of miR‐381 in human cancers. (A) The expression of miR‐381 is negatively regulated by various factors, including lncRNA, circRNA, periostin, bFGF, WISP‐1 and EZH2. (B) ADAR1 mediates the A‐to‐I editing level of miR‐381. bFGF: basic fibroblast growth factor. WISP‐1, Wnt1‐induced secreted protein‐1; EZH2, enhancer of zeste homolog 2; ADAR1, adenosine to inosine acting on RNA enzyme 1

The expression of miR‐381 is regulated by various long non‐coding RNAs (lncRNAs) (Figure 1A). In osteosarcoma, lncRNA CAT104 induces the expression of ZEB1 by sponging miR‐381, thereby increasing cell proliferation, migration and invasion and suppressing cell apoptosis.^68^ Similar effects of lncRNA CAT104 were observed in gastric cancer.^53^ The lncRNA TUG1 also functions as an endogenous suppressor of miR‐381 in gastric cancer.^16^ In cervical cancer, lncRNA TUG1 upregulates the expression of HOXA13 by inhibiting miR‐38, thereby promoting cell proliferation and invasion.^29^ In pancreatic cancer, the lncRNA DLEU1 is overexpressed and targets miR‐381 to upregulate the expression of CXCR‐4, thereby aggravating the progression of pancreatic cancer.^55^

In addition to lncRNAs, the circular RNA (circRNA) FGFR1 is involved in the regulation of miR‐381 (Figure 1A). In NSCLC, FGFR1 serves as a sponge for miR‐381 to upregulate CXCR4. The upregulation of CXCR4 by miR‐381 promotes the progression of NSCLC and induces resistance to anti‐PD‐1‐based therapy.^48^

### 4.2 Others

In addition to non‐coding RNAs, the expression of miR‐381 in cancer is regulated by several molecules (Figure 1A). Periostin is secreted by osteoblasts and indispensable for the adhesion and extension of osteoblasts.^75^ Recent studies suggest that periostin is involved in the metastatic process of human cancers. In NSCLC, periostin activates the ERK and p38 pathways, thus suppressing the expression of miR‐381.^45^ In breast cancer, enhancer of zeste homolog 2 is overexpressed and induces the resistance of breast cancer cells to cisplatin by decreasing the expression of miR‐381.^76^ In chondrosarcoma, miR‐381 is downregulated by basic fibroblast growth factor (bFGF). In osteosarcoma, WISP‐1 upregulates VEGF‐A by downregulating miR‐381, which promotes angiogenesis and deterioration of osteosarcoma.^69^

Adenosine to inosine acting on RNA enzyme 1 (ADAR1) is a regulator of miR‐381 and an oncogene in NSCLC (Figure 1B).^77^ ADAR1 catalyses adenosine‐to‐inosine (A‐ to‐I) editing, which is an essential post‐transcriptional modification that alters the sequence of the RNA molecule.^78^, ^79^, ^80^ The effect of ADAR1 on increasing tumourigenesis in NSCLC is at least partly dependent on its positive modulation of A‐to‐I editing on miR‐381.^77^ A total of 19 A‐to‐I RNA editing hotspots have been identified in 20 cancer types in a recent study and miR‐381 is one of them.^81^ Moreover, if editing occurs in the seed sequences, it can alter the targets of the microRNA, leading to the regulation of a different set of target mRNAs.^82^, ^83^ Therefore, editing of the miR‐381 seed sequence in several cancer types may shift its target mRNAs and thus its function.

## 5 CLINICAL APPLICATION POTENTIAL OF MIR‐381

### 5.1 MiR‐381 as a non‐invasive marker for cancer diagnosis and monitoring

Aberrant expression of circulating miRNAs suggests their potential as non‐invasive markers for the diagnosis of cancer. MiR‐381 is upregulated in the peripheral blood of patients with glioma.^43^ By contrast, serum miR‐381 is reduced in patients with ovarian and gastric cancers.^25^, ^84^ Plasma miR‐381 is upregulated in patients with PCa, and it is the most statistically significant circulating miRNAs.^85^ Extra efforts are supposed to be devoted to the detection of miR‐381 in blood samples.

In patients with osteosarcoma, miR‐381 expression differs between patients with postoperative recurrence and those without.^39^ The upregulation of tissue miR‐381 in patients with recurrent osteosarcoma reveals the potential of miR‐381 for the monitoring of cancer recurrence and metastasis.

### 5.2 The prognostic value of miR‐381 in cancer

Because of the differential expression of miR‐381 in different cancer types, the association of miR‐381 levels with prognosis differs among cancer patients. In patients with NSCLC, miR‐381 downregulation is related to aggressive clinicopathological characteristics, such as advanced TNM stage and lymph node (LN) metastasis.^5^, ^7^ NSCLC patients with higher tissue miR‐381 levels may have better event‐free survival and overall survival (OS) rates.^8^ Similarly, the downregulation of miR‐381 in gastric cancer is correlated with adverse clinicopathological features.^14^, ^16^ Moreover, the level of tissue miR‐381 is positively associated with progression‐free survival (PFS) and OS in patients with gastric cancer.^13^ Low expression of miR‐381 and other downregulated miRs together with the deficiency of phosphate and tension homology deleted on chromosome ten (PTEN) predicts a poor OS in patients with TNBC.^86^ In EMC, a negative association between miR‐381 expression and myometrial invasion along with LN metastasis were confirmed. The downregulation of miR‐381 in EMC may predict advanced FIGO stage.^27^

Contrary to the favourable prognostic role of miR‐381 in several cancer types, overexpression of miR‐381 predicts a poor OS in patients with osteosarcoma.^39^ In osteosarcoma patients, the miRNA profiles differ between those with pathologic fractures and those without. The combination of overexpressed miR‐381 and other dysregulated miRNAs in osteosarcoma patients with pathologic fractures is correlated with a poorer prognosis regarding the risk of metastasis and shorter OS.^87^

### 5.3 The potential of miR‐381 as a therapeutic target in cancer

In NSCLC, miR‐381 serves as the target of metformin, which is a basic diabetes medication. Treatment of NSCLC cells with metformin significantly increases the expression of miR‐381 and suppresses the expression of YAP, thereby playing an inhibitory role on cell proliferation, migration and invasion in NSCLC.^9^ MiR‐381 is also involved in the anti‐cancer activity of icaritin, a naturally active substance. Icaritin upregulates miR‐381, resulting in the downregulation of UBE2C and the suppression of cell proliferation and invasion, the arrest of the cell cycle at the G1 phase, and the induction of cell apoptosis in PCa.^63^

## 6 CONCLUSIONS AND FUTURE PERSPECTIVES

Although the role of miR‐381 in tumour biology has been researched extensively, its effect on other intracellular processes such as cell metabolism and the tumour microenvironment remains poorly understood. In addition, the complex regulatory network targeting miR‐381 is not fully elucidated in cancer. The level of circulating miR‐381 in patients with cancer needs further exploration as well to maximize the value of miR‐381 in cancer diagnosis and monitoring. In addition to the analysis of blood samples, miR‐381 detection in other biological samples, such as the bone marrow, saliva, urine and faeces, could be useful for the diagnosis and monitoring of certain cancer types, such as haematologic malignancies, OSCC, urologic neoplasms and gastrointestinal cancers. Despite extensive efforts to exploit the diagnostic and prognostic value of miRNAs, attempts to apply miRNA‐based therapies to the treatment of human cancers are rare. The therapeutic activity of certain medications targeting miR‐381 in cancer has been confirmed. However, further research is required to further investigate the potential of miR‐381‐targeted gene therapy in human cancers. In addition, the potential role of A‐to‐I editing of miR‐381 has not been studied in detail. RNA editing in miR‐381 not only decreases the “effective” amount of wild‐type miR‐381but also has the potential to inhibit a completely different set of targets. Therefore, it is important to comprehensively investigate the functional effect of miRNA editing as editing of the miR‐381 seed sequence occurs in several cancer types. Overexpression experiments in cancer cell lines including “mimics” of wild‐type and edited miR‐381 is a possible method. However, it might not represent the real tumour context. Future efforts should be undertaken to confirm the functional consequences of edited miR‐381 and investigate its clinical utility.

In conclusion, miR‐381 is involved in a variety of cancer biological functions, suggesting its potential for cancer diagnosis, prognosis and treatment. Future efforts should be devoted to the application of miR‐381‐based diagnostic and therapeutic strategies in clinical practice.

## CONFLICT OF INTEREST

The authors confirm there are no conflicts of interest.

## AUTHOR CONTRIBUTIONS

**Huanhuan Sha:** Conceptualization (lead); data curation (lead); methodology (equal); project administration (equal); resources (lead); software (lead); writing – original draft (lead); writing – review and editing (lead). **Yujie Gan:** Conceptualization (equal); formal analysis (equal); software (equal); validation (equal); visualization (equal); writing – review and editing (equal). **Feng Xu:** Data curation (equal); formal analysis (equal); writing – review and editing (equal). **Yue Zhu:** Formal analysis (equal); validation (equal). **Renrui Zou:** Formal analysis (equal); writing – review and editing (equal). **Weiwei Peng:** Validation (equal). **Zhiya Wu:** Formal analysis; visualization (equal). **Rong Ma:** Funding acquisition (lead). **Jianzhong Wu:** Writing – review and editing (equal). **Jifeng Feng:** funding acquisition (lead); investigation (equal); project administration (equal); writing – review and editing (equal).

## Data Availability

Data sharing is not applicable to this article as no new data were created or analyzed in this study.
